# Regulatory roles of epigenetic modifications in plant-phytopathogen interactions

**DOI:** 10.1007/s44297-023-00003-y

**Published:** 2023-08-10

**Authors:** Zeng Tao, Fei Yan, Matthias Hahn, Zhonghua Ma

**Affiliations:** 1 State Key Laboratory of Rice Biology, Key Laboratory of Molecular Biology of Crop Pathogens and Insects, Institute of Biotechnology, Zhejiang University, 866 Yuhangtang Road, Hangzhou, 310058 China; 2 Department of Biology, RPTU University of Kaiserslautern, PO Box 3049, 67653 Kaiserslautern, Germany

**Keywords:** DNA methylation, Histone methylation, Histone acetylation, Chromatin remodeling, Noncoding RNAs, Plant immunity, Pathogenicity, Interaction

## Abstract

As a sessile organism, plants have evolved a complex and sophisticated immune system to defend against various pathogenic microbes effectively. However, microbes have also developed complicated and delicate strategies to suppress host immunity and successfully colonize the host. Dynamic plant‒pathogen interactions require rapid and fine-tuned regulation of their gene expression. Increasing evidence has revealed that epigenetic regulation plays key roles in plant defense-related transcriptional reprogramming, as well as microbe pathogenicity. In this review, we summarize and highlight the current progress in understanding the roles of epigenetic regulation and factors, including DNA/RNA modification, histone modification, chromatin remodeling and noncoding RNAs, in plant immunity, phytopathogen pathogenicity and their interactions. We also discuss that epigenetic regulation emerges as an efficient strategy for crop breeding and plant disease control.

## Introduction

As sessile organisms, plants have always suffered from various pathogenic microbes. To effectively resist their invasion, plants have evolved a complex and sophisticated immune system. The “zig zag” model describes a classical plant immune process, which is divided into two relatively independent and interplayed levels, including pathogen-associated molecular patterns (PAMPs) to stimulate pattern-triggered immunity (PTI) defense responses and pathogen-derived effector-triggered immunity (ETI). PTI and ETI do not function independently but have synergistic effects that amplify each other to ensure that plants can output a durable and strong immune response against the invasion of pathogens [1, 2]. ETI is regulated by the pattern recognition receptor (PRR) of PTI, indicating that ETI is dependent on PTI. Furthermore, activation of ETI promotes the transcription of resistance genes such as *WRKY31* and *ICS1* (*ISOCHORISMATE SYNTHASE 1*) and extends the phosphorylation status of BIK1 (Botrytis-Induced Kinase 1) and MPK3 (Mitogen-Activated Protein Kinase 3), which implies that ETI can enhance PTI [1, 2]. Activation of PTI and ETI mainly leads to the occurrence of a series of early immune responses, including calcium influx via channels, reactive oxygen burst and MAPK cascade activation in PTI, hypersensitive response and programmed cell death in ETI, changes in immune-related hormones in plants and transcriptional reproduction of downstream resistance genes. PRRs and intracellular nucleotide-binding leucine-rich repeat (LRR) proteins (NLR) are master regulators of immune cascades that are also associated with the expression of downstream defense genes [1, 3]. In turn, pathogens have evolved various strategies, such as activation of pathogenesis-associated genes, to inhibit host immunity and successfully invade hosts [4, 5]. Mounting evidence has shown the importance of epigenetics in the transcriptional regulation of genes involved in plant‒pathogen interactions [6, 7]. Broadly speaking, epigenetics refers to stimuli-triggered changes in gene expression due to processes that arise independent of changes in the underlying DNA sequence [8]. These processes mainly include DNA/RNA methylation, histone modifications, chromatin remodeling, and noncoding RNAs. In this review, we revisit the recent literatures on the roles of epigenetic factors in the modulation of plant‒pathogen interactions. Since the interaction systems studied thus far are very diverse, we focus on the interaction of plants with oomycetic and fungal pathogens. In addition, we were also interested in discussing potential strategies developed based on epigenetics for plant disease management.

## Basic mechanisms of epigenetic modifications in regulating gene transcription

### DNA modifications

**Fig. 1:**
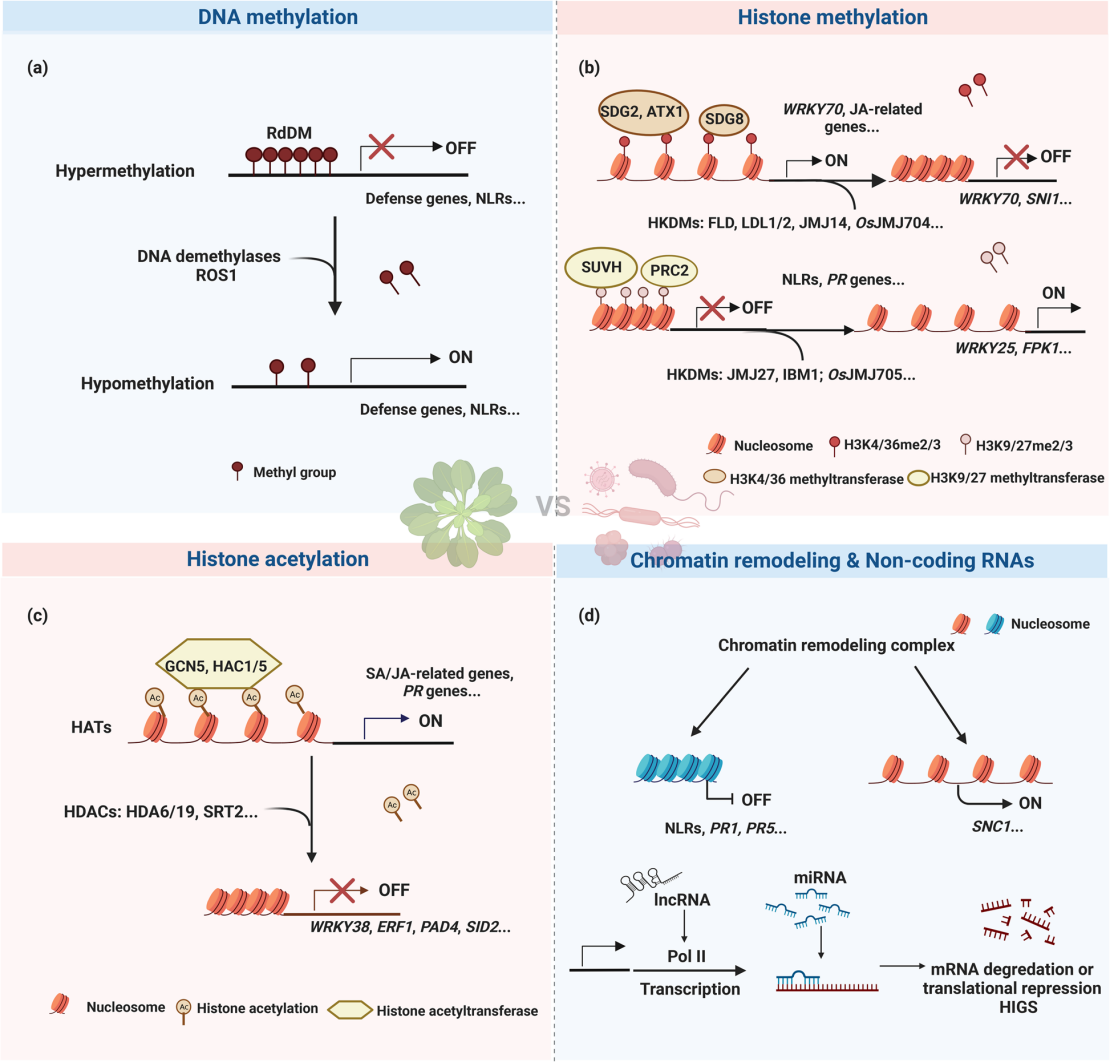
Regulatory roles of epigenetic modifications in plant immunity. **a** DNA methylation and DNA demethylation. DNA methylation usually leads to gene silencing. When the DNA methylation level is low or unmethylated, the gene is expressed normally. Conversely, transcription is inhibited. **b** Histone methylation and histone demethylation. Depending on the histone lysine sites and methyl numbers, it may lead to activation or repression of gene transcription. H3K4me2/3 and H3K36me2/3 are associated with activating gene transcription, while H3K9me2/3 and H3K27me2/3 inhibit gene transcription. **c** Histone acetylation and histone deacetylation. Histone acetylation usually results in transcriptional activation, while histone deacetylation is accompanied by transcriptional suppression. **d** Chromatin remodeling and noncoding RNAs. The chromatin remodeling complex utilizes the energy from ATP hydrolysis to drive nucleosome sliding along DNA, thus regulating the accessibility of transcription factors or DNA-binding proteins to DNA. NcRNAs are involved in plant metabolism, growth and development, stress response and other biological processes. This diagram was created with BioRender.com. The genes without the *Os* species prefix in the figure represent *Arabidopsis* genes, and gene names plus the *Os* species prefix represent genes from rice (*Oryza sativa*)

DNA modification is a basic marker of epigenetic modification and plays critical roles in gene imprinting, stress response, plant growth and development, and plant immunity [9]. The most fundamental DNA modification is the methylation of the carbon-5 of cytosine (5mC), which contributes to gene expression, transposon silencing, chromosome interaction and trait inheritance at the molecular level [10]. It occurs in different sequence contexts, including symmetrical CG, CHG, and asymmetrical CHH for transcriptional silencing (where H = A, T, or C) [11, 12]. Usually, the CG context occurs predominantly in methylated gene bodies, while transposable elements (TEs) are methylated in all three sequence contexts. In plants, three processes are involved in DNA methylation: de novo DNA methylation, methylation maintenance, and DNA demethylation [11]. De novo DNA methylation is established by a specific RNA-directed DNA methylation (RdDM) pathway and two plant-specific RNA polymerases, Pol IV and Pol V. Maintenance of DNA methylation means that some of the methylation sites can change from the copied hemimethylated sequence to the full methylated sequence by the action of methyltransferase during cell proliferation, and it depends on the composition of cytosine sequences and is catalyzed by DNA methyltransferases regulated by different mechanisms [10]. For example, maintenance of CG, CHG, and CHH occurs through METHYLTRANSFERASE 1 (MET1), CHROMOMETHYLASE 3 (CMT3) and CMT2, and CMT2 or RdDM, respectively, in *Arabidopsis*. DNA demethylation is achieved by DNA demethylase such as REPRESSOR OF SILENCING 1 (ROS1), DEMETER (DME), DEMETER-LIKE 2 (DML2), and DML3 to remove 5-methyl cytosine and replace it with unmethylated cytosine and inhibit hypermethylation at genomic locations to allow proper levels of gene expression [11, 12]. In addition to 5mC, adenine N6-methylation (6 mA) is another prominent DNA modification that is associated with either activating or suppressing gene transcription [13] (Fig. 1).

### RNA modifications

Chemical modification of RNA was found to be ubiquitous across plants and serves as an emerging important epigenetic modification in plant immunity regulation. RNA modification plays an important role in the regulation of gene expression, both at the transcriptional and posttranscriptional levels [14]. There are 160 types of RNA modifications characterized so far, most of which occur in transfer RNA (tRNA) and ribosomal RNA (rRNA) [15, 16]. N6-methyl-adenosine (m6A) is one of the most common RNA modifications in eukaryotes and has recently been implicated as a novel epigenetic marker that is involved in various biological processes [13]. The m6A modification is also a reversible chemical modification that is regulated by methyltransferases and demethylases, as well as “reader” proteins, which are specific RNA binding proteins that recognize the methylation state and specifically bind the m6A methylated region [17]. RNA m6A modification is involved in various kinds of RNA regulation, including mRNA stability, translation efficiency, RNA splicing, nuclear export, and RNA structure [13]. Through highly dynamic regulation of RNA methylation and demethylation, organisms reprogram their transcriptome and maintain their genome stability in a rapidly changing environment. Since these processes are closely related to many important agronomic traits, applying RNA modification effectively will open a new perspective for improving agronomic traits in plant breeding [18].

### Histone modifications

Histone modifications are common markers of epigenetic regulation in plants. Posttranslational modifications (PTMs) of histones include methylation, acetylation, phosphorylation, ubiquitination, and SUMOylation, in which different kinds or amounts of PTMs in the same or different histone sites, such as the “histone code”, coordinate or antagonize to form chromatin [19]. Histone methylation often occurs on lysine (K) or arginine (R) residues of H3 and H4 histones. Histone methylation is catalyzed by histone methyltransferases (HMTs), removed by histone demethylases (HDMs), and recognized by “reader” proteins [20, 21]. Histone lysine methylation can be mono-, di-, and trimethylated, and common sites include H3K4, K9, K36, K79, and H4K20. Depending on the sites and numbers of lysine modifications, histone methylation is usually associated with either activating or repressing gene transcription [22]. For example, H3K4me2/3 (di/tri-methylation of lysine 4 on histone H3) and H3K36me2/3 are associated with activating gene transcription, while H3K9me2/3 and H3K27me2/3 inhibit gene transcription.

Histone acetylation/deacetylation is another well-characterized histone modification that is dynamically regulated by histone acetyltransferases (HATs) and histone deacetylases (HDACs) [23]. HATs catalyze acetylation of lysine (K) residues, which is associated with loosening of the chromatin structure and results in transcriptional activation, whereas histone deacetylation has the opposite effect of inhibiting transcription. Histone acetylation also occurs at various sites, such as H3K4, H3K9, H3K27, H3K36, H3K79, H4K5, H4K12, H4K16 and H4K20. Three families of conservative HATs, namely, the p300/CBP, GCN5/PCAF and MYST families, are present [23, 24]. At the same time, four major families of HDACs exist: Class I (HDAC1/2/3/8), Class II (HDAC4/5/6/7/9/10), Class III (SIRT1-7) and Class IV (HDAC11), in which classes I, II and IV depend on Zn^2+^, and Class III requires NAD^+^ to produce nicotinamide [4, 25]. Although different modifications are catalyzed or removed by their specific enzymes, these modifications coordinately contribute to regulating gene transcription in the same chromatin context [26]. Above all, accumulated researches have revealed that these histone codes play fundamental roles in plant‒pathogen interactions [24, 27] (Fig. 1).

### Chromatin remodeling

The nucleosome is the basic unit of chromatin in eukaryotes and is assembled in a highly compressed state in a confined space in the nucleus [28]. To successfully read the information encoded by DNA, chromatin has evolved highly complex and exquisite dynamic regulation, which mainly acts through the chromatin remodeling complex [29]. ATP-dependent chromatin remodeling complexes (CRCs) utilize the energy from ATP hydrolysis to drive nucleosome sliding along DNA, to alter nucleosome occupancy and positioning, and/or to mediate nucleosome removal and replacement by variant histones, thus regulating the accessibility of transcription factors or other DNA-binding proteins to DNA [30]. According to the structural characteristics of their catalytic subunits, the existing chromatin remodeling complex subfamilies can be roughly classified into four categories: SWI/SNF, ISWI, CHD and INO80/SWR1 [31]. By establishing a chromatin structure for the transcriptional machinery, CRCs play an integral role in regulating gene expression in concert with histone and DNA modifiers, variant histones, histone chaperones, and other transcriptional regulators. CRCs are involved in the regulation of DNA replication, transcription, recombination repair, and plant immunity [31] (Fig. 1).

### Noncoding RNAs

Noncoding RNAs (ncRNAs) refer to the general term of ribonucleic acids, which are not encoded as proteins but directly perform biological functions in the form of RNAs [32]. According to their origin, biogenesis and mechanism of action, ncRNAs can be classified into two categories: housekeeping ncRNAs and regulatory ncRNAs. Housekeeping ncRNAs include transfer RNAs (tRNAs), small nuclear RNAs (snRNAs), ribosomal RNAs (rRNAs) and small nucleolar RNAs (snoRNAs), while regulatory ncRNAs are composed of microRNAs (miRNAs), small interfering RNAs (siRNAs), PIWI-interacting RNAs (piRNAs) and long noncoding RNAs (lncRNAs) [33]. Although regulatory ncRNAs cannot be translated into proteins, they play vital regulatory roles in different ways. miRNAs usually collaborate with siRNAs to specifically cut target transcripts to silence gene expression or inhibit the translation of target genes by restricting the recruitment or movement of ribosomes [34]. Moreover, miRNAs and siRNAs also mediate DNA and histone methylation to regulate gene expression [35, 36]. LncRNAs can regulate the expression of their target genes through *cis*/trans-action and interactions with chromatin remodeling complexes [37–40] (Fig. 1).

## Epigenetic regulation in plant immunity

### DNA/RNA modification

In plants, widespread dynamic DNA methylation occurs during pathogen infection, which is significantly associated with defense responses. By profiling the DNA methylomes of plants treated with bacterial pathogens or SA, numerous differentially methylated regions associated with differentially expressed genes were detected [41–43]. Moreover, mutants that are defective in global maintenance of DNA methylation show enhanced resistance to *Pseudomonas syringae pv. tomato* DC3000 in *Arabidopsis* [41]. In addition, global DNA hypomethylation, especially CHH methylation, was observed in rice and tomato upon treatment with flg22, which triggered the downregulated expression of numerous RdDM-related genes [44]. A null allele of the putative Pol IV second subunit, NRPD2, encodes the second largest subunit of plant-specific RNA polymerases IV and V (Pol IV and Pol V), which are crucial for the RdDM pathway. Pol V-defective mutants, but not Pol IV mutants, show enhanced disease resistance towards the bacterial pathogen *Pst* DC3000 [45]. ARGONAUTE4 (AGO4), which directs DNA methylation in the RNA-directed DNA methylation (RdDM) pathway, is required for resistance to *Pst* DC3000 in *Arabidopsis* [46].

REPRESSOR OF SILENCING 1 (ROS1) encodes an active DNA demethylase that promotes basal resistance towards *Pst* DC3000 [47]. ROS1 acts on the disease resistance gene *RESISTANCE METHYLATED GENE 1* (*RMG1*) by catalyzing demethylation in its promoter [47]. Furthermore, ROS1 directly binds to the promoters of *RMG1* and *ORPHAN RECEPTOR LIKE PROTEIN 43* (*RLP43*) to facilitate DNA demethylation during flg22 induction, thus promoting binding to *RLP43* by WRKY transcription factors and activating its transcription [47]. Triple mutant of DNA demethylase *rdd* (ros1 dml2 dml3) increased susceptibility to the fungal pathogen *Fusarium oxysporum* [48]. In addition, DNA demethylases can positively regulate the expression of stress-responsive genes enriched with transposon or repeat sequences in their promoter regions for defense against fungal pathogens [48]. Many downregulated genes in *rdd* are also downregulated in the RdDM mutants *nrpd1* and *nrpe1*, which show enhanced susceptibility to *F. oxysporum* infection [48].

Several resistance (R) proteins or nucleotide-binding leucine-rich repeat receptor (NLR) genes are clustered in regions of TEs and highly repetitive sequences enriched with DNA methylation and H3K9me2 for adaptive genome defense [6, 49]. DNA methylation was reported to be involved in the transcriptional regulation of NLR genes during plant‒pathogen interactions. PigmS, a rice NLR receptor, suppresses PigmR-mediated broad resistance by interfering with PigmR homodimerization [50]. The expression of *PigmS* was associated with DNA methylation, as two tandem transposons were present in the promoter region of *PigmS*. The lower level of DNA methylation in the transposons increased transcription of *PigmS* and further compromised PigmR-mediated resistance [50]. Thus, this strategy associated with epigenetic regulation elegantly enables rice with the NLR receptor to confer durable resistance to the fungus *Magnaporthe oryzae* without yield penalty [50]. DNA methylation present in the promoter of *Pib* plays a novel enhancing role in conditioning a high level of induced expression in times of rice blast infection, and it confers resistance to the pathogen [51].

Accumulating evidences have revealed that m6A is involved in the modulation of many plant biological processes, such as embryo development [52, 53], floral transition [54], circadian clock and metabolism [55], as well as plant‒pathogen interactions. The first m6A methyltransferase identified in mammals was named METTL3 and was successfully cloned from a 200 kDa methyltransferase complex. It is a member of the S-adenosine-L-methionine (SAM)-dependent methyltransferase family, which is highly conserved in plants and mammals [56]. It can be phosphorylated and activated by a key kinase of antiviral pathways to increase mRNA stability and protein translation, which leads to secure antiviral immunity [57]. m6A is involved in wheat resistance to infection by the RNA virus WYMV (wheat yellow mosaic virus) [58]. A genome-wide association study in *Triticum aestivum* revealed that the susceptibility (S) gene encoding m6A methyltransferase B (*Ta*MTB) is a positive regulator of WYMV infection. Upon infection, *Ta*MTB is translocated from the nucleus into cytoplasmic aggregates to upregulate the m6A level of WYMVRNA1 by direct binding and stabilize the viral RNA, thus promoting viral infection [59]. Moreover, some defense-responsive genes were modified with m6A to increase their expression after bacterial fire blight (*Erwinia amylovora*) inoculation in pear (*Pyrus bretschneideri*) [60]. m6A significantly correlated with the immune response to CGMMV (cucumber green mottle mosaic virus) infection in watermelon [61].

### Histone methylation and demethylation

H3K4 methylation is highly conserved and catalyzed by COMPASS (Complex proteins associated with Set1) [62]. Nine SET DOMAIN GROUP (SDG) proteins, which encode histone lysine methyltransferases with conserved SET domains [63], have been reported to affect H3K4 methylation in *Arabidopsis*, namely, ATXR3/SDG2, ASH1-RELATED3 (ASHR3/SDG4), ARABIDOPSIS TRITHORAX-RELATED7 (ATXR7/SDG25), ASH1-RELATED PROTEIN1 (ASHH1/SDG26), ARABIDOPSIS TRITHORAX 1 (ATX1/SDG27), ATX2 (SDG30), ATX3 (SDG14), ATX4 (SDG16), and ATX5 (SDG29) [64]. H3K4 modification is usually associated with transcriptional activation [62]. Related studies have shown that elevated levels of H3K4me3 at many disease-resistance genes enhance plant immunity. SDG2 is a major H3K4 methyl transferase in *Arabidopsis,* and loss of SDG2 leads to increased sensitivity to *Botrytis cinerea* [20]. WRKY70, a regulator of SA and JA signaling, is required for systemic acquired resistance (SAR) and acts downstream in the defense signaling pathway. The transcription of *WRKY70* was found to be regulated by the histone methyltransferase ATX1 [65] (Table 1).

**Table 1:** Epigenetic regulators in plant immunity

Sites	Modification	Species	Modifier	Activation/Repression	Affected Genes	References
H3K4	Methylation	*Arabidopsis thaliana*	SDG2	Activation	JA-Related Genes	[20]
		*Arabidopsis thaliana*	ATX1	Activation	WRKY70	[65]
	Demethylation	*Arabidopsis thaliana*	FLD	Repression	SAR-Related Genes	[66]
		*Arabidopsis thaliana*	LDL1/2	Repression	WRKY22/40/70	[67]
		*Arabidopsis thaliana*	JMJ14	Repression	SNI1	[68]
		*Oryza sativa L*	JMJ704	Repression	Bacteria Blight-Related Genes	[69]
H3K36	Methylation	*Arabidopsis thaliana*	SDG8	Activation	NLRs-Related Genes	[70]
H3K9	Methylation	*Arabidopsis thaliana*	SUVH4/5/6	Repression	NLRs-Related Genes; PR1	[71]
	Demethylation	*Arabidopsis thaliana*	JMJ27	Activation	WRKY25; PRs Genes	[72]
		*Arabidopsis thaliana*	IBM1	Activation	FPK1; PRs Genes	[73]
H3K27	Methylation	*Arabidopsis thaliana*	MEA	Repression	JA-Related Genes	[74]
		*Arabidopsis thaliana*	LHP1	Repression	JA/SA/ET-Related Genes	[75]
		*Hordeum vulgare*	PRC2	Repression	Powdery Mildew-Responsive Genes	[76]
	Demethylation	*Oryza sativa L*	JMJ705	Activation	*Xanthomonas*-Responsive Genes	[77]
Histone Acetylation		*Arabidopsis thaliana*	GCN5	Activation	SA/JA-Related Genes	[78]
		*Arabidopsis thaliana*	HAC1/5	Activation	PRs Genes; SA-Related Genes	[79]
Histone deacetylation		*Arabidopsis thaliana*	HDA6	Repression	PR1/2; WRKY38	[80]
		*Arabidopsis thaliana*	HDA19	Repression	ERF1; WRKY38/62	[81]
		*Arabidopsis thaliana*	SRT2	Repression	PAD4; SID2	[82]
Chromatin remodeling factors	SWI/SNF subfamily	*Arabidopsis thaliana*	SWP73A	Repression	NLRs	[83]
		*Arabidopsis thaliana*	SYD	Repression	SNC1	[84]
	INO80 subfamily	*Arabidopsis thaliana*	CHR19	Repression	JA/SA-Related Genes	[85]
		*Arabidopsis thaliana*	PIE1	Repression	SAR-Mediated Genes	[86]
		*Arabidopsis thaliana*	ARP6	Repression	PTI-Mediated Genes	[86]
		*Arabidopsis thaliana*	NRP	Activation	SA-Related Genes	[87]
		*Arabidopsis thaliana*	SWC6	Activation	PR1/5	[88]
	ISWI subfamily	*Arabidopsis thaliana*	CHR11	Repression	Defense-Responsive Genes	[89–91]
	CHD subfamily	*Arabidopsis thaliana*	CHR5	Activation	SNC1	[92]

H3K36 methylation is generally considered an active marker for intergenic regions and is enriched in intragenic regions. SDG4/8/26 contain conserved AWS, SET, and post-SET domains, which have been shown to be responsible for H3K36 methylation [64]. SDG8 plays a crucial role in plant defense against fungal pathogens by regulating a subset of genes within the jasmonic acid (JA) and ethylene signaling pathways and NLR-mediated pathogen resistance in *Arabidopsis* [93, 94]. Loss of *SDG8* resulted in faster emergence of the hypersensitive response (HR) to DC3000 strains of *P. syringae* [93]. SDG25 and SDG8 regulate pep1-, flg22-, effector-triggered immunity and systemic acquired resistance by affecting CCR2- and CER3-specific histone lysine methylation. Moreover, *sdg8* and *sdg25* mutants show increased sensitivity to *B. cinerea* and *A. brassicicola* [95]*.*

H3K9 methylation is associated with silenced regions in the genome. In *Arabidopsis*, 15 proteins (SUVH1-10, SUVR1-5) were proposed to regulate H3K9 methylation. KYP/SUVH4, SUVH5, and SUVH6 are responsible for maintaining H3K9 methylation; SUVH4 mediates the majority of H3K9me2 methylation in both constitutive and facultative heterochromatin, while SUVH5 and SUVH6 play only minor roles in H3K9me2 methylation [96]. It has recently been reported that SUVH1, SUVH3, SUVH7, and SUVH8 play a role in transcriptional activation but not silencing [97]. Recently, the distribution of H3K9me2, which is established by the H3K9 methyltransferase SUVH4, was shown to trigger programmed cell death (PCD) in response to immune- and disease-promoting pathogen effectors [98, 99]. The H3K9 methyltransferase SUVH4/5/6 was reported to facilitate defense priming of NLR genes by direct occupancy at promoters to prevent their constitutive activation. Under normal growth conditions, *suvh4/5/6* maintains basal *PR1* repression and displays more resistance to *Pst* DC3000 than wild-type *Arabidopsis* [71].

H3K27 methylation is catalyzed by evolutionarily conserved polycomb repressive complex 2 (PRC2), which plays a key role in the formation of facultative heterochromatin and transcriptional silencing [100, 101]. PRC was first discovered in *Drosophila melanogaster* to silence the homeobox gene and is conserved in most eukaryotes, which is composed of PRC2 and PRC1 [101]. PRC2 is mainly responsible for the catalytic modification of H3K27me2/3, consisting of three core subunits, Kmt6, Suz12, and Eed, and one accessory subunit, RbAp48/Nurf55, where KMT6 encodes H3K27 methyltransferase. PRC1, including Pc, Ph and Psc, is responsible for the monoubiquitination of histone H2A and compact adjacent chromatin, thus achieving stable transcriptional silencing [100, 101]. H3K27me3 occupies large genomic regions that regulate many genes in *Arabidopsis*, and the maintenance of H3K27me3 is largely independent of DNA methylation or RNA interference [102]. Recently, a study revealed that H3K27me3 and H3K4me3 modifications work together to affect stress-responsive gene expression in response to powdery mildew in hulless barley. H3K27me2 is mainly distributed in the facultative heterochromatin region. H3K27 mono-methyltransferases, *ARABIDOPSIS TRITHORAX-RELATED PROTEIN5* (*ATXR5*) and *ATXR6*, lead to rereplication of specific genomic locations, having pleiotropic effects on plant development. In *Arabidopsis*, MEDEA (MEA), CURLY LEAF (CLF), and SWINGER (SWN) are three H3K27 methyltransferases of PRC2. A recent study showed that *MEA* is epigenetically silenced via DNA methylation in the vegetative stage, whereas its transcription is upregulated with pathogen infection and in turn limits pathogen growth in plants. Thus, loss of *MEA* resulted in increased resistance to *B. cinerea* and *Pst* DC3000, while overexpression of *MEA* resulted in increased susceptibility. Furthermore, MEA interacts with the transcription factor DROUGHT INDUCED 19 (DI19) and recruits the *RPS2* locus to inhibit its transcription, thus attenuating the defense response in *Arabidopsis* [74]. Knockout of *LHP1* (*LIKE HETEROCHROMATIN PROTEIN 1*), a PRC1 component, results in reduced levels of salicylic acid (SA) and increased susceptibility to *Pst* DC3000 [75].

In addition to histone methyltransferases, histone demethylases also play an important role in plant immunity. In *Arabidopsis* and rice, FLOWERING LOCUS D (FLD), LDL1-3 and JmjC domain-containing proteins (JMJs) can reduce the levels of histone H3-Lys 4 methylation in chromatin [103–105]. RSI/FLD codes for putative histone-demethylase and influences the H3K4me3 modification level of *WRKY29* and *WRKY6* promoters [106]. FLD function is also required for priming *WRKY38*, *WRKY65* and *WRKY53* and activating SAR in distal tissue [106]. Systemic SA fails to accumulate in the *fld* mutant when infected with pathogens, which indicates that FLD is essential for SAR in *Arabidopsis* [107]. GLUTATHIONE-STRANSFERASE THETA 2 (GSTT2), a member of the glutathione-S-transferase theta class, interacts with FLD and affects the level of H3KAc and H3K4me2/3 at the promoters of *WRKY6* and *WRKY29* genes [108]. *LDL1* and *LDL2*, two homologues of human LYSINE-SPECIFIC DEMETHYLASE-LIKE 1 (LSD1), function redundantly and suppress the immune response against *Pst* DC3000 infection, which may be caused by H3K4me2/3-dependent downregulation of *WRKY22/40/70* genes [67]. JmjC domain-containing proteins JMJ14-18 can also demethylate not only mono- and di-methylated but also trimethylated substrates [109]. JMJ14, an H3K4 demethylase, plays a crucial role in modulating local and systemic defense responses [68]. As JMJ14 negatively modulates H3K4me3 levels of *SNI1* (*Suppressor of NPR1-1 Inducible 1*), a negative regulator of SAR, loss of *jmj14* results in susceptibility to the virulent pathogen *Pst* DC3000 [68]. JMJ27, an H3K9me1/2 demethylase, is involved in the defense response by negatively regulating *WRKY25* and positively regulating *PR* genes. INCREASE IN BONSAI METHYLATION 1 (IBM1), encoding H3K9me2 demethylase, plays a critical role in immunity by modulating the transcription of *PR1*, *PR2*, and *FRK1* [73]. However, a recent study reported that *ibm1* mutants showed increased resistance to *Pst* DC3000 [110]. In rice, JMJ707 suppressed the expression of negative regulators, such as *NRR*, *OsWRKY62* and *Os-11N3*, by reducing their level of H3K4me2/3 at promoters. *OsJMJ704* is involved in H3K4me2/3 demethylation and is a positive regulator of bacterial blight resistance in rice [69]. *OsJMJ705*, an H3K27me2/3 demethylase, removes H3K27me3 from defense-related genes and is involved in plant immunity during pathogen infection. Overexpression of *OsJMJ705* enhanced resistance to rice bacterial blight disease, while loss of *JMJ705* resulted in enhanced susceptibility [77]*.* Another H3K27me3 demethylase *RELATIVE OF EARLY FLOWERING 6* (*REF6*), together with *HEAT SHOCK TRANSCRIPTION FACTOR A2* (*HSFA2*), forms a positive feedback loop to attenuate immunity in *Arabidopsis* [111]*.*

### Histone acetylation and deacetylation

In general, a higher level of histone acetylation is associated with an open chromatin state and active transcription [112]. General control non‐repressed protein 5 (GCN5) participates in the histone acetylation module of the Spt-Ada-Gcn5 acetyltransferase (SAGA) complex. GCN5 acetylates lysine 14 of histone 3 (H3K14ac), H3K9ac and H3K27ac in the promoter regions of its targets [113–115]. Recent studies suggest that GCN5 participates in salicylic acid (SA)-mediated immunity by regulating H3K14ac levels at the 5’ and 3’ ends of targeted genes. Specifically, dysfunction of *GCN5* leads to decreased levels of H3K14ac at the 5’ end of downregulated genes and an increase at the 3’ end of upregulated genes [116]. Intriguingly, recent studies have demonstrated that GCN5 plays a vital role in JA-mediated resistance against *B. cinerea* by modulating nonhistone acetylation. In the absence of JA, GCN5 can strengthen the interaction of two corepressors, TOPLESS (TPL) and NOVEL-INTERACTOR-OF-JAZ (NINJA), and promote their recruitment to *MYC2* targets by enhancing TPL acetylation, which facilitates downstream transcriptional repression. With JA treatment, transcription of *HDA6* is transiently induced, leading to transcriptional activation of *MYC2* by decreasing the acetylation level and repressor activity of TPL, which maintains homeostasis in a reversible manner [78]. Moreover, HACs, the CBP/p300-family histone acetyltransferases, form a coactivator complex with NONEXPRESSOR OF PATHOGENESIS-RELATED GENES 1 (NPR1) and bind to the chromatin of *PATHOGENESIS-RELATED GENES* (*PRs*) through TGACG SEQUENCE-SPECIFIC BINDING PROTEIN (TGA) transcription factors, which lead to the development of SA-triggered immunity and *PR* induction [79]. In wheat, the histone acetyltransferase *Ta*HAG1 was defined as a positive regulator of powdery mildew resistance. *Ta*HAG1 physically interacts with *Ta*PLATZ5, a plant-specific zinc-binding protein, which can directly bind and regulate the expression of the key transducer gene *TaPAD4* and promote SA and ROS (reactive oxygen species) accumulation to render resistance to *Bgt* infection [117] (Table 1).

Histone lysine acetylation is a reversible covalent modification that is removed by histone deacetylases (HDACs). Tremendous progress has been made in revealing the function of HDACs in many biological processes, including embryonic development, defense response, and genome stability [25, 112]. HDAC6, an RPD3/HDA1-type deacetylase in *Arabidopsis*, represses the defense-responsive genes *PR1*, *PR2* and *WRKY38* by removing H3Ac levels, subsequently compromising plant defense against *Pst* DC3000 [80]. HDA6 directly binds to the SA biosynthesis genes *CALMODULIN BINDING PROTEIN 60 g* (*CBP60g*) and *SYSTEMIC ACQUIRED RESISTANCE DEFICIENT 1* (*SARD1*) and suppresses their expression by decreasing the levels of histone acetylation, thus regulating SA-mediated plant immunity [118]. In addition to HDA6, HDA19 is another RPD3-like HDAC in *Arabidopsis *[119]. Dysfunction of *HDA19* leads to compromised resistance, whereas its overexpression results in enhanced resistance. Overexpression of *HDA19* results in increased expression of *ETHYLENE RESPONSE FACTOR 1* (*ERF1*) and *Basic Chitinase* (*CHI-B*) and displays resistance against the pathogen *Alternaria brassicicola* [81]. HDA19 can also physically interact with the transcriptional activators WRKY38 and WRKY62 to abolish their negative regulation of immunity [120]. HDA9 was reported to interact with the WD40-repeat protein HOS15 (High Expression of Osmotically Responsive Genes 15) to regulate NLR genes, likely by reducing H3K9Ac, leading to susceptibility to *Pst* DC3000 [121]. In wheat, *Ta*HDA6 and *Ta*HOS15 are involved in a histone deacetylase complex to directly bind and suppress defense-related genes such as *TaPR1*, *TaPR2*, *TaPR5*, and *TaWRKY45*, resulting in suppression of wheat powdery mildew resistance [122]. Another histone deacetylase SRT2, a homolog of yeast Sir2, negatively regulates plant basal defense against the pathogen *Pst* DC3000 in *Arabidopsis*. Disruption of *SRT2* leads to increased expression of the SA biosynthesis genes *PAD4*, *EDS5* and *SID2*, thereby enhancing resistance [82].

### Chromatin remodeling

Chromatin remodeling complexes (CRCs) are involved in regulating a variety of biological processes, including plant immunity [123]. In the absence of pathogens, SWI/SNF-ASSOCIATED PROTEINS 73 (SWP73A), a subunit of the SWI/SNF CRC, acts as a transcription suppressor and directly binds to the promoters of *RPS2,* *ZAR1*, and *RPP1*-like genes to suppress their expression, avoiding autoimmunity [124]. Upon *Pst* DC3000 infection, miR3440 and siRNA-*SWP73A* were induced to target *SWP73A* and downregulate its expression, thus activating the plant immune response [83]. SPLAYED (SYD), an SWI/SNF chromatin remodeler, represses *SUPPRESSOR OF NPR1* (*SNC1*), which encodes a NOD-LIKE RECEPTOR (NLR) protein in *Arabidopsis*, at the transcriptional level [84]. Furthermore, SYD was also revealed to regulate the expression of multiple defense genes of JA/ET signaling pathways and plays a critical role in resistance against *B. cinerea* [125]. CHROMATIN REMODELING 19 (CHR19), a subunit of ATPase of the INO80 subfamily, utilizes ATP to slide nucleosomes and causes substantial changes in genome-wide nucleosome positioning and occupancy [85]. Under normal growth conditions, CHR19 represses numerous SA/JA stress-responsive genes and regulates plant resistance to different pathogens, whereas loss of *CHR19* results in higher susceptibility to the fungal pathogen *B. cinerea* [85]. SWR1 is involved in a highly conserved INO80 subfamily group, which acts as a scaffold and core catalytic subunit to assemble the SWR1-C complex. SWR1-C has four subunits, including PHOTOPERIOD-INDEPENDENT EARLY FLOWERING1 (PIE1), ACTIN-RELATED PROTEIN6 (ARP6), SWR1 COMPLEX 6 (SWC6) and H2A. Z [88]. PIE1, SWC6 and H2A. Z are positive regulators of resistance against both biotrophic and necrotrophic pathogens, while ARP6 is a negative regulator of defense against biotrophic pathogens [86]. Furthermore, *PIE1* is involved in the regulation of crosstalk among different defense signaling pathways [88]. Histone chaperone NUCLEOSOME ASSEMBLY PROTEIN-RELATED PROTEIN (NRP) interacts with SWR1-C to regulate H2A. Z deposition, which is a conserved variant of classical histone H2A and affects transcription by regulating nucleosome conformation [87, 126]. Loss of *NRP* is more sensitive to infection with *Pst* DC3000, while overexpression of *NRP* increases resistance to the pathogen [127]. In addition, SWR1-C and H2A. Z may participate in plant resistance to different pathogens in a temperature-dependent manner [128]. *CHR11* and *CHR17*, two functionally redundant genes, encode ISWI proteins in *Arabidopsis*. ICHR11 and CHR17 repress a large number of defense-responsive genes and negatively regulate plant disease resistance [89–91]. CHR5 is a positive regulator of both basal and NLR-mediated resistance to bacterial pathogens by regulating nucleosome occupancy in the promoter regions of *SNC1* [92] (Table 1).

### Noncoding RNAs

With the development of high-throughput sequencing technology, a large number of ncRNAs have been identified and explored that are widely involved in plant metabolism, growth and development, stress response and other biological processes [129]. In particular, ncRNAs play an important role in plant resistance to phytopathogen invasion. First, RNA silencing in plants is known as posttranscriptional gene silencing (PTGS) or coinhibition, which allows plants to resist the invasion of foreign nucleic acids (transposons, transgenes or viruses) and protects the integrity of their genome. The system is effective for plants in antiviral defense, similar to other defense systems and in keeping with the “arms race” concept of host‒pathogen interactions, in which viruses encode one or more RNA-silencing suppressors to resist host RNA-silencing-mediated defense responses [130]. Next, to prevent inappropriate or excessive immunity of plants, miRNAs and secondary siRNAs directly target NLRs to avoid their inappropriate expression in the absence of infection [131]. Finally, it has been found that host RNA can inhibit fungal growth by targeting key genes, a phenomenon known as host-induced gene silencing (HIGS), which is the first evidence of RNA transboundary transfer from plants to fungi [132, 133]. For example, two cotton miRNAs, miR159 and miR166, are induced by *Verticillium dahliae* infection and directly target virulence-related genes to silence their transcription to suppress invasion [134]. HIGS has also been demonstrated in a variety of pathogens, including *Blumeria graminis*, *Fusarium verticillioides*, *Botrytis cinerea* and the oomycetes *Bremia lactucae*, *Phytophthora infestans* and *Phytophthora capsica* [135–139]. The discovery of this transkingdom RNA silencing has enabled the development of RNA-based approaches to control a wide range of crop pests and diseases [129].

## Epigenetic regulation in filamentous pathogen pathogenicity

### DNA/RNA modification

DNA methylation was revealed to widely regulate fungal development and mycotoxin biosynthesis. Fungal plant pathogens predominantly possess four types of DNA MTase homologues, namely, DIM-2, DNMT1, DNMT5, and RID [140]. DNA methylation mainly occurs in transposable elements (TEs), gene promoter regions, and repetitive DNA sequences in fungi [140, 141]. In *M. oryzae*, which causes rice blast disease, approximately 20% of non-TE genes are also methylated in the mycelia, and methylation is frequently found near the start and end of coding regions and distant from the center. Deletion of DNA methyltransferase indicated that proper reprogramming of DNA methylation is required for asexual reproduction in the fungus [142]. Moreover, RNA-seq analysis showed that DNA methylation is associated with transcriptional silencing of transposable elements and transcript abundance of genes in a context-dependent manner, reinforcing the role of DNA methylation as a genome defense mechanism [142]. DNA methylation and histone modification usually coordinate to regulate fungal development and mycotoxin biosynthesis. In the saprophytic fungus *Neurospora crassa*, deposition of 5mC is mediated by the DNA methyltransferase (DNMT) DIM-2. Moreover, DIM-2 targeted H3K9me3-marked genomic loci through a direct interaction with heterochromatin protein 1 (HP1), thus facilitating the methylation of nearby DNA loci [143] (Table 2).

**Table 2:** Epigenetic regulators in microbe pathogenicity

Sites	Modification	Species	Modifier	Activation/Repression	Affected Genes	References
H3K4	Methylation	*Magnaporthe oryzae*	SET1	Activation	Pathogenicity related genes	[144]
	Demethylation	*Botrytis cinerea*	KDM5	Repression	ROS production related genes	[145]
H3K36	Methylation	*Saccharomyces cerevisiae*	SET2	Activation	Growth-related genes	[146]
		*Magnaporthe oryzae*	KMT2H	Activation	Conidium development and pathogenicity related genes	[147]
H3K9	Methylation	*Botrytis cinerea*	DIM5	Repression	Pathogenicity related genes	[148]
		*Epichloë festucae*	KMT1	Repression	Symbiosis-specific genes	[149]
	Demethylation	*Neurospora crassa*	LSD1	Activation	Pathogenicity related genes	[150]
H3K27	Methylation	*Fusarium graminearum*	PRC2	Repression	Pathogenicity related genes	[151, 152]
		*Magnaporthe oryzae*	PRC2	Repression	Growth-related genes	[153, 154]
		*Ustilaginoidea virens*	PRC2	Repression	Secondary metabolites related genes	[155]
Histone Acetylation		*Fusarium graminearum*	GCN5	Activation	Autophagy related genes; ATG8	[156]
		*Fusarium graminearum*	SAS3	Activation	DON production related genes	[42]
		*Fusarium graminearum*	ELP3	Activation	Virulence related genes	[157]
		*Magnaporthe oryzae*	GCN5	Activation	Autophagy related genes; ATG7	[158]
		*Magnaporthe oryzae*	SAS3	Activation	Pathogenicity related genes	[159]
		*Magnaporthe oryzae*	HAT1	Activation	Autophagy related genes; ATG3/9	[160]
Histone deacetylation		*Fusarium graminearum*	HDF1	Repression	Pathogenicity related genes	[161]
		*Fusarium graminearum*	FNG1	Repression	Pathogenicity related genes	[162]
		*Magnaporthe oryzae*	HDA1/HOS2	Repression	Vegetative growth and conidiation related genes	[163]
		*Magnaporthe oryzae*	TIG1	Repression	Pathogenicity related genes	[164]
		*Magnaporthe oryzae*	SNT2	Repression	Autophagy related genes	[165]
		*Magnaporthe oryzae*	RPD3/HST4	Repression	Pathogenicity related genes	[166]
		*Magnaporthe oryzae*	SIN3	Repression	Pathogenicity related genes	[167]
		*Magnaporthe oryzae*	SIR2	Repression	A superoxide dismutase gene	[168]
		*Fusarium fujikuroi*	HDA1/2	Repression	Secondary metabolism and virulence related genes	[169]
Chromatin-remodeling factors	SWI/SNF subfamily	*Fusarium graminearum*	SWP73	Repression	CYP51A; DNA damage response related genes	[170]
		*Fusarium graminearum*	ARP9	Repression	CYP51A	[170]
	ISWI subfamily	*Verticillium dahliae*	*Vd*Dpb4	Repression	DNA damage response related genes	[171]

N6-methylation (6 mA) methyltransferases that modulate patterns of 6 mA marks across the genome were discovered in the oomycetes *Phytophthora infestans* and *Phytophthora sojae* [172]. Methylated DNA is depleted around the transcription start site (TSS) and enriched with weakly expressed genes, particularly transposon elements, which implies that 6 mA may be associated with genomic adaptive evolution [172]. Furthermore, N6-methyladenosine (m6A), which is abundant on mRNA, plays key roles in the regulation of RNA function. *Mo*MTA1, a putative methyltransferase, was revealed to be involved in m6A modification and autophagy for fungal infection in *M. oryzae* [173]. RNA enriched with m6A peaks was negatively related to their abundance in vivo. Deletion of *Mo*MTA1 resulted in 659 hypomethylated m6A peaks covering 595 mRNAs, and 114 of these m6A peaks were negatively related to mRNA abundance, including several *ATG* transcripts involved in autophagy. At the same time, deletion of *MoMTA1* showed reduced virulence due to blockage of appressoria penetration and invasive growth, as well as severely disordered autophagy [173].

### Histone methylation and demethylation

H3K4 methylation is usually associated with activating transcription, in which H3K4me1 is highly enriched in the enhancer region, and H3K4me2/3 is enriched at the transcriptional initiation sites and gene body [21]. In *Saccharomyces cerevisiae*, COMPASS is composed of the catalytic subunits Set1, Swd1-3, and Bre2 [21, 174]. In *M. oryzae*, components of COMPASS, such as Bre2, Spp1, and Swd2, which are homologous to yeast COMPASS subunits, are all required for *Mo*Set1-catalyzed H3K4 methylation [144]. Deletion of *SET1* and other components caused similar defects regarding invasive hyphal development and pathogenicity, as H3K4me3-marked genes are often involved in spore germination and pathogenesis in *M. oryzae* [144, 175]. In *Fusarium graminearum*, Set1, together with Bre2, SPP1 and Swd2, mediates H3K4 methylation, which plays critical roles not only in the regulation of fungal growth and secondary metabolism but also in multiple stress responses [176]. The transcription factor AreA regulates putrescine-mediated transcription of TRIs by facilitating the enrichment of H3K4 me2/3 and histone H2B monoubiquitination (H2B ub1) on TRIs, whereas H2B ub1 regulates H3K4 me2/3 via the COMPASS component *Fg*Bre2 in *F. graminearum* [177]. In *Fusarium fujikuroi*, H3K4me2/3 is enriched at gibberellic acid (GA) biosynthesis clusters to activate GA-associated gene expression, increase GA biosynthesis and contribute to fungal pathogenicity [178]. Moreover, H3K4me3 occupied the conidiation-specific transcription factor *Aba1* to increase fungal conidiation [178]. In addition, the H3K4 demethylase KDM5 was also identified in *F. fujikuroi* and *B. cinerea* [145]. *Bc*KDM5 controls ROS production and affects proper assembly of the septin protein and virulence to initiate infection structure formation and host penetration. Loss of *BcKDM5* impairs conidiation, appressorium formation and stress adaptation and abolishes infection cushion formation and virulence [145] (Table 2).

H3K36 methylation activates transcription and is enriched in intergenic and intragenic chromosomal regions [146]. In *S. cerevisiae*, there is a single methyltransferase, Set2, that catalyzes H3K36 [21]. In addition to being enriched in gene bodies to activate gene transcription, H3K36me2 can also recruit DNA methyltransferase 3A (Dnmt3A) and histone deacetylase Rpd3 to inhibit cryptic transcription, which is contrary to the role of H3K36me2/3 in activating gene transcription [146]. In *N. crassa*, there are two H3K36 methyltransferases, Set2 and Ash1 [179]. Set2 is responsible for catalyzing the majority of H3K36me3 methylation to mediate transcriptional activation, whereas Ash1 catalyzes H3K36me2 for transcriptional repression. Moreover, deletion of either *SET2* or *ASH1* results in abnormal fungal growth [179]. In *M. oryzae*, deletion of *SET2* and *Ash1*-like genes was reported to have no effect on H3K36me2/3 accumulation, but both resulted in abnormal fungal growth, conidium development and pathogenicity [147, 175]. In *F. fujikuroi*, Set2 is responsible for H3K36 methylation in euchromatic regions, while Ash1 catalyzes H3K36 at the subtelomeric regions, including the accessory chromosome [180]. Recently, a study in *P. sojae* also revealed that *Ps*KMT3, encoding H3K36 methyltransferases, is required for asexual development and pathogenicity [181].

H3K9 methylation is associated with the formation of constitutive heterochromatin and plays a key role in maintaining genome stability. Clr4 (KMT1 complex) in *S. pombe* and Dim5 in *N. crassa* were responsible for catalyzing the methylation of H3K9. Normal genome-wide H3K9me distribution is essential for both pathogens and symbionts in fungi–host interactions [143]. In *B. cinerea*, loss of *DIM5* results in nearly abolished H3K9me3 and causes downregulation of pathogenicity genes associated with host signal sensing, host tissue colonization, stress response, toxin synthesis, and response to host immunity [148]. In the plant endosymbiotic fungus *Epichloë festucae*, H3K9me3 catalyzed by Clr4 (KMT1), together with H3K27me3, is required for the transcription of symbiosis-specific genes associated with the biosynthesis of loliterms and ergot alkaloids, which are silenced under nonsymbiotic culture conditions [149]. These genes are activated by the removal of H3K9me3 when the fungus interacts with the plant host [149]. Deletion of Clr4 led to severe defects in colony growth and hyphal development, as well as attenuated pathogenicity [182]. In *N. crassa*, the conserved histone demethylase LYSINE-SPECIFIC DEMETHYLASE 1 (LSD1) regulates heterochromatin by removing both H3K4me and H3K9me [150]. Deletion of LSD1 results in a hyper-H3K9me3 phenotype, but the spread of H3K9me3 is dependent on the presence of DNA methylation, as well as histone deacetylation [150].

Polycomb repressive complexes, including PRC2 and PRC1, exist in mammals and higher plants; however, no components of PRC1 have been found in lower eukaryotes such as fungi. H3K27me3 has a positive effect on fungal growth and pathogenicity [174]. PRC2 and H3K27me3 modification were first identified in *N. crassa* [183]. H3K27me3 occupied 6.8% of the regions in all chromosomes, and H3K27me3-marked genes are rarely related to development; thus, knockout of the PRC2 component has little effect on its growth and development in *N. crassa* [183]. However, deletion of PRC2 components not only seriously impaired fungal growth and pathogenicity but also led to the accumulation of secondary metabolites in *F. graminearum* [151, 152]. In *M. oryzae*, deletion of PRC2 components also resulted in abnormal growth and weakened pathogenicity [153, 154, 175]. In another rice pathogenic fungus, *Ustilaginoidea virens*, H3K27me3 was also shown to have a conservative role in fungal development, virulence and production of secondary metabolites [155].

Notably, the silencing mechanisms deployed on effector genes are well understood to be associated with H3K27me3 occupancy in pathogenic fungal genomes [184]. Effector proteins and their transcriptional regulation play important roles in the infection process of pathogenic fungi [184]. They are usually distributed in the subtelomere regions where heterochromatin and transposon elements are enriched in the *Phytophthora* genome [184]. In *Zymoseptoria tritici*, the chromatin regions of effector genes, such as *AvrStb6* and *Avr3D1*, were enriched with H3K27me3 modification, resulting in their transcriptional silencing in the vegetative growth stage [185]. In *P. sojae*, the effector gene *Avr1b* was also transcriptionally silenced with H3K27me3 occupation in the mycelia; thus, the resistance of soybean root rot mediated by the resistance gene *Rps1b* was not expressed [186]. In *M. oryzae* and *U. virens*, numerous effector genes were enriched with H3K27me3 modification to maintain transcriptional silencing in the vegetative growth stage, whereas the majority of these effectors were transcriptionally activated *in planta* [153–155]. However, the molecular mechanism of how to achieve derepression of effector genes during host infection is still unclear.

As a hallmark of facultative heterochromatin, H3K27me3 plays key roles in transcriptional reprogramming of the targeted gene. For example, genes encoding secondary metabolites and effectors in pathogenic fungi are transcriptionally silenced in association with H3K27me3 enrichment in the vegetative stage but activated *in planta* [154]. It is necessary to reveal how H3K27me3-mediated facultative heterochromatin is built and stable transcriptional silencing is achieved without the existence of PRC1 in fungi. The diversity and complexity of H3K27me3-regulatory mechanisms is always a hotspot in the epigenetics community. Recently, a conserved BAH–PHD-containing protein, BP1, was identified as a reader protein that elegantly revealed how methylation of H3K27 was recognized in the chromatin in *F. graminearum* [152]. An additional subunit of P55 and histone deacetylase component were discovered and required for H3K27me3 building and stable transcriptional silencing in *M. oryzae* [167].

### Histone acetylation and deacetylation

HATs such as GNAT, MYST, and the p300/CBP family, which regulate the transcription of pathogenic genes and acetylate nonhistone proteins, have been well studied in pathogenic microbes [187]. In *F. graminearum*, GCN5 of the SAGA histone acetyltransferase complex plays an important role in fungal development and plant infection by regulating the expression of target genes. Deletion of *FgGCN5* results in reduced perithecium formation, increased sensitivity to oxidative and osmotic stresses, and loss of production of the mycotoxin deoxynivalenol [42]. Interestingly, alterations of histone acetylation through inhibiting the activity of *Fg*GCN5 could be triggered by the interacting bacteria from its host microbiome, thus repressing gene expression and suppressing fungal growth and pathogenicity in *F. graminearum* [156]. The histone acetyltransferase *Fg*SAS3 is also essential for DON production and pathogenicity in wheat head infection [42]. Elongator protein 3 (ELP3), a member of the GNAT family, is involved in sexual and asexual development, virulence, and oxidative stress response in *F. graminearum* [157]. *Fg*SAS3 is indispensable for the acetylation of histone site H3K4 and important for fungal morphogenesis, DON biosynthesis, and pathogenicity [42]. *Mo*SAS3, the catalytic subunit of the MYST histone acetyltransferase complex, is also required for development and pathogenicity in *M. oryzae* [159] (Table 2).

Importantly, HATs also have the ability to modify nonhistone proteins through acetylation. In fungi, autophagy and its homeostasis play essential roles in fungal growth and virulence. In *M. oryzae*, MoGcn5 negatively regulates light and nitrogen starvation-induced autophagy by acetylating the autophagy-related protein Atg7 in the cytoplasm [158]. MoHat1 acetylates the autophagy-related proteins Atg3 and Atg9 to orchestrate appressorium formation and pathogenicity [158, 160]. In *F. graminearum*, Gcn5 negatively regulates fungal autophagy by acetylating the autophagy-related protein Atg8 and blocking its cellular relocalization [188].

The catalytic subunits of HDACs are generally divided into three major classes in fungi: class I, Rpd3 and Hos2; class II, Hda1 and Hos3; and class III, Sirt1, Sir2, Hst1, Sirt3/Hst4, Sirt4, and Sirt5 [189]. In *M. oryzae*, treatment with the HDAC inhibitor trichostatin A inhibited appressorium differentiation and pathogenicity [190]. and are required for vegetative growth and conidiation and appressorium formation [163]. The *M. oryzae* Tig1-HDAC complex is required for vegetative growth, conidia production, and pathogenicity [164]. Hos2 interacts with Snt2, as the core component of the Tig1 complex, and deacetylates H3K18 and H4K16 [163–165]. Moreover, Snt2 was found to regulate autophagy and infection by regulating the acetylation state of histone H3 and transcription of autophagy-related genes [165]. In addition, Rpd3 and Hst4 differentially regulate mycelial growth, asexual development, and pathogenesis in *M. oryzae* [191]. Rpd3 was able to functionally complement the *RPD3* gene deletion mutant in yeast [166], and knockdown of the *RPD3* gene caused defects in asexual and sexual reproduction as well as conidial germination and appressorium formation rates and a significant reduction in pathogenicity in *M. oryzae*, whereas overexpression of *RPD3* led to increased conidia formation, decreased production of infection hyphae, and loss of pathogenicity [166, 191]. As a component of the same Rpd3-HDAC complex, deletion of *switch-independent 3* (*SIN3*) caused severely restricted mycelial growth and abnormal asexual development [167, 191]. Notably, Sin3 was required to sustain H3K27me3 occupancy and stably maintain gene repression by directly interacting with P55, an accessory subunit of PRC2 [167]. Histone deacetylase Sir2 inhibits the host immune response by regulating the expression of a superoxide dismutase gene and is critical for the invasive *in planta* growth of *M. oryzae* [168]. In *F. graminearum*, deletion of *HDF1*, an orthologue of *HOS2*, resulted in a significant reduction in virulence and deoxynivalenol production and deficiency in sexual reproduction and conidiation [161]. *Fg*Fng1, a component of the Rpd3-HDAC complex, acts antagonistically with histone acetyltransferase Esa1 on histone H4 acetylation, which is important for vegetative growth, conidiation, sexual reproduction, and plant infection [162]. Similarly, in *F. fujikuroi*, Hda1 and Hda2 are important for secondary metabolism and virulence [169].

### Chromatin remodeling

In addition to DNA methylation, RNA modification, and histone modification, chromatin remodeling plays an equally important role in regulating the expression of pathogenic genes in pathogenic microorganisms. In *F. graminearum*, the chromatin remodeling factor *Fg*SWI/SNF proteins Swp73 and Arp9 interact with phosphorylated *Fg*SR, a transcription factor that controls sterol biosynthesis, and subsequently facilitate transcriptional activation of the ergosterol biosynthetic gene *CYP51A*. Moreover, deletion of Arp9 caused transcriptional repression of *CYP51A* by increased nucleosome occupancy at its promoter [170]. Moreover, the *Fg*SWI/SNF protein Swp73 was further shown to be involved in activating the expression of DNA damage response-related genes. The SWI/SNF complex was also recruited by *Fg*AreB, a pioneer transcription factor in the nitrosative stress (NS) response, at the promoters of genes involved in the NS response, thus promoting their transcription [192]. *Vd*Dpb4, a conserved component of the yeast ISW2 complex in *V*. *dahliae*, encoding a histone-fold protein of the ISW2 chromatin remodeling complex, facilitates DNA damage repair in response to plant ROS stress in *Verticillium dahliae*, a soil-borne pathogenic fungus that causes vascular wilt in a wide range of plants. Deletion of *Vd*Dpb4 resulted in a more compact chromatin structure and affected the ATP-dependent chromatin-remodeling factor ISW2-dependent transcriptional effect on gene expression, including genes involved in DNA damage repair [171] (Table 2).

### Noncoding RNAs

NcRNA-mediated RNA silencing closely affects the virulence of filamentous pathogens. Disruption of the core components of RNA silencing affects the mycelial growth, spore development and pathogenicity of the pathogen. Deletion of DR1 or AGO3 in *M. oryzae* leads to reduced hyphal growth and virulence to the host [193]. Mutants of *BcDCL1* or *BcDCL2* show reduced virulence and delayed growth [194]. Mutants of *VdAGO1* and *VdAGO2* are defective in hyphal growth [195]. Moreover, transkingdom RNA silencing in the reverse direction can affect the infection of pathogens. It was found that RNAs from pathogens could be transferred to plant cells to promote their own infection. For example, *Bc*-siR3.1, *Bc*-siR3.2, and *Bc*-siR5 can be transferred to infected *Arabidopsis* cells to silence host defense genes [194]. Oomyces *Hyaloperonospora arabidopsidis* sRNAs can be incorporated into AGO1 proteins in plants [196]. *Phytophthora infestans* can also transfer its sRNAs to infected plants and target mRNAs that are beneficial for plant immunity [197].

## Epigenetic regulation in plant‒pathogen interactions

Epigenetic regulation is involved in priming defense genes for faster and stronger transcription. Plant immune responses are often promoted to a primed state of enhanced defense. Defense priming is established in tissue exposed to pathogen-associated molecular patterns (PAMPs), microbe-associated molecular patterns (MAMPs), herbivore-associated molecular patterns (HAMPs), damage-associated molecular patterns (DAMPs), effectors, or chemical compounds and in the systemic, unharmed, or untreated parts of the plant [66, 198, 199]. Therefore, primed plants show more rapid and robust activation of defense responses when challenged by pathogens, insects, or abiotic stress, and this is frequently associated with local and systemic immunity and stress tolerance. These defense-priming processes include systemic acquired resistance (SAR), which is induced by necrotizing pathogens and requires salicylic acid (SA) and pipecolic acid (PA). The induced systemic resistance (ISR), which is activated by growth-promoting bacteria and fungi, depends on jasmonate (JA) and ethylene (ET) [200]. H3K4me3 was reported to be uncoupled from defense gene expression [201]. Expression of *WRKY29* is associated with H3K4me3, H3K4me2, and acetylation of H3K9 (H3K9ac), H4K5ac, H4K8ac, and H4K12ac, which are systemically induced by treatment with the resistance inducer benzothiadiazole. This might create a memory of the primary infection that is associated with an amplified reaction to a second stress stimulus [201]. Treatment of common bean with two other resistance inducers, β-aminobutyric acid (BABA) and 2,6-dichloroisonicotinic acid (INA), induced plant resistance to *P. syringae pv. phaseolicola* infection and changed the levels of H3K4me3 and H3K36me3 in defense-related genes [202].

In the long evolution of dynamic plant‒pathogenpathogen interactions, pathogen effectors compel the evolution of plant mechanisms that link pathogen sensing to rapid and effective defense activation to minimize fitness costs [184, 203]. In plant‒pathogen interactions, transcriptional regulation of effector genes from pathogens is often used as a powerful weapon to manipulate host immunity [184]. Pathogen effectors can suppress the activation of defense genes by interfering with host epigenetic factors during infection. The cytoplasmic effector *Ps*Avh23, produced by the soybean pathogen *P. sojae*, has the capability of binding to the ADA2 subunit of the HAT complex and interferes with the association of Ada2 with the catalytic subunit Gcn5 [204]. Subsequently, *Ps*Avh23 suppresses H3K9 acetylation mediated by the Aad2/Gcn5 module and increases plant susceptibility. Moreover, ectopic expression of *PsAvh23* resulted in decreased H3K9 acetylation levels at the corresponding loci and misregulation of defense-related genes [204]. Effector protein UvSec117 in *U. virens* targets the histone deacetylase *Os*HDA701 from rice and negatively regulates rice broad-spectrum resistance [205]. Secretion of UvSec117 enhanced *Os*HDA701-modulated deacetylation, accompanied by reduced levels of H3K9ac and suppressed expression of defense-responsive genes in rice. Furthermore, host-induced gene silencing of *UvSec117* promotes rice resistance to *U. virens* [205]. The *P. sojae* effector *Ps*Avh52 was found to be physically associated with the soybean transacetylase GmTap1 and facilitated GmTap1 relocation into the nucleus. GmTap1 acetylated histones H2A and H3 during early infection, thereby promoting susceptibility to *P. sojae*, while GmTap1 remained confined to the cytoplasm and did not modify plant susceptibility in the absence of *Ps*Avh52 [206]. Thus, epigenetic control seems to mediate the transcriptional plasticity of effectors, which could be a robust strategy to enable the adaptation of pathogens to their host.

Not only can regulators from pathogens suppress plant immunity by interfering with host epigenetic factors, but plants can also inhibit the virulence of pathogens when fighting against them. Recently, researchers have found that plants can also transfer their own RNAs to pathogens, therefore affecting their pathogenicity. MicroRNA sequencing and northern blot analysis showed that tissues of cotton inoculated with *V. dahliae* contained miR166, miR159 and other plant-derived miRNAs, indicating that specific host miRNAs can be exported into the fungal hyphae [134]. Moreover, miR166 and miR159 were predicted to target *Clp-1* (Ca2 + -dependent cysteine protease) and *HiC-51* (isotrichodermin C-15 hydroxylase), which are two virulence genes of *V. dahliae*. After mutating the target sites of microRNAs in the two genes, their expression levels were significantly upregulated in the infected cotton, suggesting that the expression levels of the two genes were decreased under normal conditions, possibly because miR166 and miR159 transferred to the fungal hyphae and inhibited the expression of their target genes. Subsequent inoculation experiments showed that miR166 and miR159 could participate in host resistance to pathogens by targeting and silencing the expression of *Clp*-*1* and *HiC*-*51* [134]. *Arabidopsis* cells can secrete exosome-like extracellular vesicles to transfer sRNAs to *Botrytis cinerea* to inhibit its pathogenicity [207]. Subsequent experiments proved that exosomes could be effectively taken up by *B*. *cinerea.* TET8 (TETRASPANIN–like 8) and TET9 are two exosome markers upon *B*. *cinerea* infection. Double mutants of *tet8 tet9* obtained by artificial miRNA are more sensitive to *B*. *cinerea,* and sRNA levels of the host plant were lower in the isolated *B*. *cinerea* from the double mutant, supporting that TET8 and TET9 mediate the transfer of sRNAs of *Arabidopsis* to enhance plant resistance to *B*. *cinerea*.

## Conclusion and perspective

In this review, we highlighted the importance and detailed mechanisms of epigenetic regulation in plant‒pathogen interactions. It is becoming clear that the antagonistic activity of epigenetic modification writers and erasers facilitates the dynamic regulation of gene expression. These different modifications are highly interconnected and orchestrate transcriptional reprogramming of plant immunity, microbe pathogenicity, and their interactions. The coordinated functions of crosstalk with different kinds of epigenetic modifications, such as that between DNA methylation and histone modifications and that between histone methylation and acetylation, remain to be addressed in the given chromatin contexts.

Plants have evolved sophisticated mechanisms to adapt to fluctuating environments, including immune systems, to deal with diverse infectious microbes. Epigenetic variations respond to pathogen infections and can be harnessed by pathogenic effectors, thereby increasing plant phenotypic plasticity and resources for disease improvement. In response, plant pathogens have also developed a substantial degree of phenotypic plasticity to avoid and/or suppress recognition by the host. Such dynamic interactions compel the evolution of plant mechanisms that link pathogen sensing to rapid and effective defense activation to minimize fitness costs. To date, studies on plant‒pathogen interactions have usually focused on the chromatin dynamics of plants to adapt and defend against pathogenic microbes. Little information has been revealed on how pathogenic microbes sense, adapt and invade their plant hosts, especially in terms of coordinating their chromatin dynamics.

For plant disease management, efforts to understand the roles of epigenetics in the modulation of plant‒pathogen interactions would provide opportunities to explore novel strategies for disease management. Since epigenetic modifications are vital for various cellular processes of plant pathogens, exploration of epigenetic modification inhibitors may have great potential in developing new drugs with novel modes of action for agricultural applications. For instance, Chen et al. identified a natural active compound (phenazine-1-carboxamide) secreted by the biocontrol agent *Pseudomonas piscium* that directly binds and affects the activity of the fungal histone acetyltransferase GCN5, leading to deregulation of histone acetylation at H2BK11, H3K14, H3K18, and H3K27 in *F. graminearum*, as well as suppression of fungal growth, virulence, and mycotoxin biosynthesis [188].

For resistance breeding, the mechanisms of epigenetic regulation are widely used in crop disease resistance strategies. Chemical inhibitors, as well as resistance inducers, have been applied to increase crop disease resistance. For example, BABA, the chemical inducer of SAR and defense priming, was found to enhance broad-spectrum resistance to various pathogens, including hemibiotrophic bacteria, necrotrophic fungi and oomycete pathogens, by regulating the levels of histone modifications, such as H3K4me2/3 or H3K36me3, to affect the transcription of their target genes in plants [202, 208, 209]. BABA has been widely used to improve disease resistance in some vegetables and fruits [202, 208, 210–213]. Additionally, CRISPR/dCas9-based epigenetic strategies to manipulate targeted gene expression would be a powerful tool for future crop design [214]. Previously, this method only targeted the removal of 5mC at specific loci in the genome with high specificity, which could develop new epialleles for traits of interest and reactivate the expression of previously silenced genes, transgenes, or transposons [215]. Recently, the methods were further developed and applied in other silencing pathways that suppressed gene expression through either DNA methylation or histone H3K27me3 deposition, H3K4me3 demethylation, histone deacetylation, and inhibition of RNA polymerase II transcription [216]. CRISPR/dCas9-based epigenetic strategies will provide a more comprehensive understanding of epigenetic regulatory pathways in plants and provide an armament of tools for targeted gene manipulation in crop resistance breeding.
